# Nutritional, Phytochemical, and Biological Characterization of Peel, Pulp, and Seed Powder from the Fruits of *Berberis mikuna* and *Berberis burruyacuensis*: Potential as a Functional Ingredient

**DOI:** 10.3390/plants14101418

**Published:** 2025-05-09

**Authors:** Enzo Agustín Matteucci, María Eugenia Orqueda, Mariana Leal, María Inés Isla, Mario Simirgiotis, Iris Catiana Zampini, Oscar R. Dantur, María Alejandra Moreno

**Affiliations:** 1 Instituto de Bioprospección y Fisiología Vegetal (INBIOFIV-CONICET-UNT), San Miguel de Tucumán T4000CBG, Tucumán, Argentina; matteucci_agustin@hotmail.com (E.A.M.); eorqueda@yahoo.com.ar (M.E.O.); maariileal@hotmail.com (M.L.); misla@csnat.unt.edu.ar (M.I.I.); zampini@csnat.unt.edu.ar (I.C.Z.); 2 Facultad de Ciencias Naturales e IML, Miguel Lillo 205, San Miguel de Tucumán T4000, Tucumán, Argentina; 3 Instituto de Farmacia, Facultad de Ciencias, Universidad Austral de Chile, Campus Isla Teja, Valdivia 5090000, Chile; mario.simirgiotis@uach.cl; 4 Farm “Las Nubes”, Altos de Medina, Burruyacú T4117XAS, Tucumán, Argentina; odantur@gmail.com

**Keywords:** *Berberis mikuna*, *Berberis burruyacuensis*, nutritional composition, phytochemical composition, functional properties

## Abstract

*Berberis mikuna* Job. (common name “mikuna”) and *Berberis burruyacuensis* O.R. Dantur, S. Radice, E. Giordani and Papini (common name “sacha mikuna”) are endemic native plant species from northwestern Argentina. The aim of this work was to evaluate, for the first time, the potential of the pulp, seed, and peel powders from *B. mikuna* and *B. burruyacuensis* fruits as functional food ingredients, with the purpose of adding value to these native resources and promoting their sustainable use. All powders exhibited nutritional value due to their protein, lipid, fiber, and ash content, especially the seed powder. Phenolic compounds (including xanthone, phenolic esters, coumarins, flavonoids, tannins, and anthocyanins), alkaloids, amino acids, lipids, and vitamins, totaling 33 compounds, were identified in the pulp, seed, and peel of both *Berberis* fruits through UHPLC-PDA-ESI-QT-MS/MS. High anthocyanin content was observed in the pulp and peel, mainly in *B. mikuna* (195.55 ± 7.75 and 283.49 ± 6.55 g C3GE/100 g of powder, respectively), while tannins were abundant in the seeds (3.64 ± 0.11 and 6.09 ± 0.06 mg PB2/100 g of powder for *B. mikuna* and *B. burruyacuensis*, respectively). The powders exhibited antioxidant activity (ABTS^•+^; H_2_O_2_) and the capacity to inhibit enzymes related to metabolic syndrome, such as α-glucosidase, α-amylase, and lipase. These findings suggest the potential of *B. mikuna* and *B. burruyacuensis* fruit powders as functional food ingredients, dietary supplements, or natural functional colorants for foods and beverages.

## 1. Introduction

**Figure 1:**
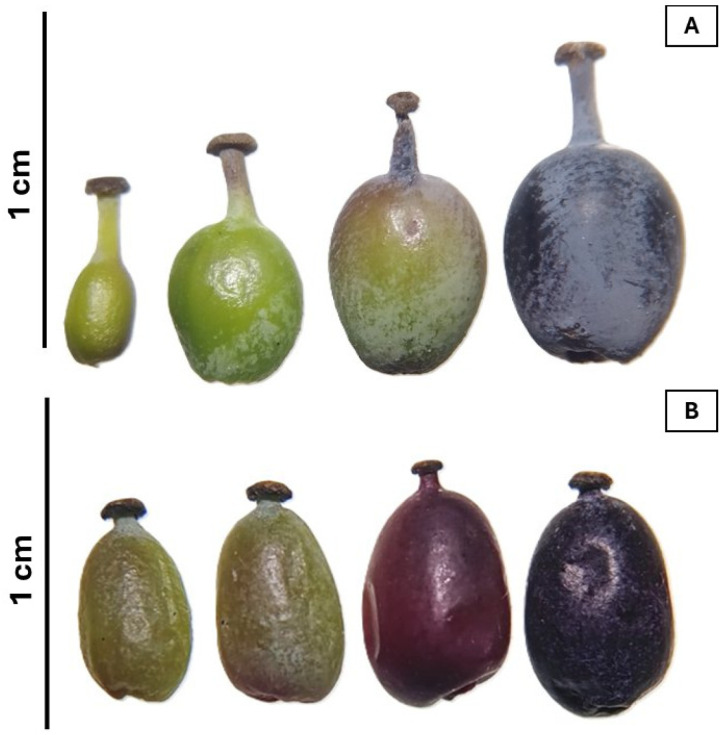
(**A**) *Berberis mikuna* and (**B**) *Berberis burruyacuensis* fruits at different maturation stages.

*Berberis* is the genus of the Berberidaceae family with the greatest number of species, totaling around 500, which are distributed throughout the globe. On the American continent, they extend from the north of the USA to the south of the Patagonian Andes of Argentina and Chile [1]. Approximately 26 species are recognized in Argentina and are distributed in two main areas, the Patagonian steppe and subantarctic forest and the Tucuman–Bolivian subtropical forest [2,3,4,5,6]. *Berberis mikuna* Job. (common name “mikuna”) and *Berberis burruyacuensis* O.R. Dantur, S. Radice, E. Giordani and Papini (common name “sacha mikuna”) are wild berry species endemic in the northwest of Argentina (NWA) [7]. Their dark purple or black fruits are edible (Figure 1), such as *Berberis microphylla*, known as calafate, and *Berberis darwinii* (michay). *B. burruyacuensis* fruits are smaller than those of *B. mikuna* (Figure 1). They are consumed fresh or prepared as jellies, marmalades, and wines [8]. *B. mikuna* was used by the Diaguita Calchaqui community as a dye, and today, their stem and roots are used by the “Warmi Pura” group in Tucumán (Argentina) and others for dyeing wool and natural fibers. Berberine, primarily stored in the roots and stem, is the alkaloid responsible for the yellow dye [8]. Most *Berberis* species have recognized medicinal properties due to the presence of berberine, berbamine, and phenolic compounds, as well as nutritional value, which is attributed to their fruits [3,9,10,11,12,13,14,15]. A previous study demonstrated that the fruits of *B. mikuna* from NWA exhibit high concentrations of anthocyanins and total phenols, comparable to those found in *B. microphylla* fruits from Tierra del Fuego, Argentina [16]. To date, there are no reports on the phytochemistry composition of *B. burruyacuensis* fruits or descriptions of the biological activities of the fruits of both species.

In this context, the objective of this study was to assess the nutritional and functional properties of the peel, pulp, and seed powders of *B. mikuna* and *B. burruyacuensis* fruits with the aim of enhancing the value of these native resources and promoting their sustainable and comprehensive utilization.

## 2. Results and Discussion

**Figure 2:**
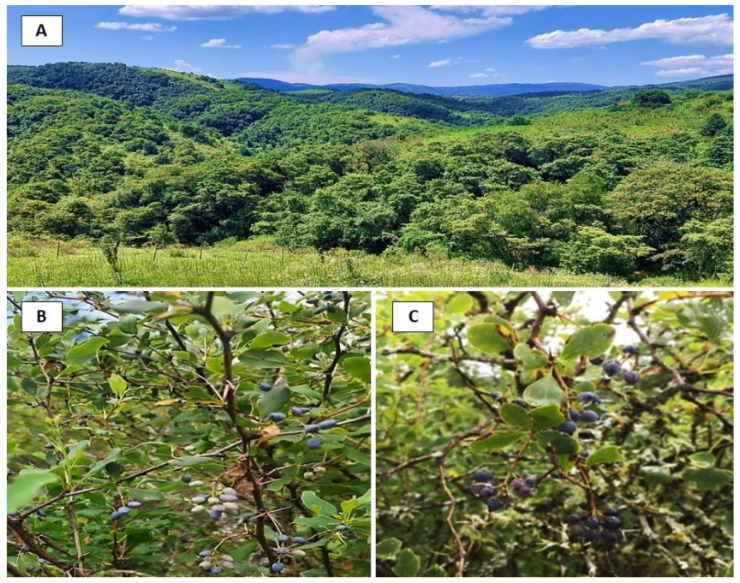
(**A**) Altos de Medina, Burruyacú, Tucumán. (**B**) *Berberis mikuna* and (**C**) *Berberis burruyacuensis* plants.

This study utilized *B. mikuna* and *B. burruyacuensis* fruits collected from Altos de Medina, located at 1500 m.a.s.l. in the Burruyacú department, Tucumán, Argentina (Figure 2). The pulp, peel, and seeds were separated, dried to a constant weight, and ground into fine powders. These powders were analyzed for their chromatic characteristics and nutritional composition. Ethanolic extractions were obtained from each part of the fruit (seeds, pulp, and peel), which were chemically characterized, and their functional properties were evaluated.

### 2.1. Chromatic Parameters of Berberis Powder

**Figure 3:**
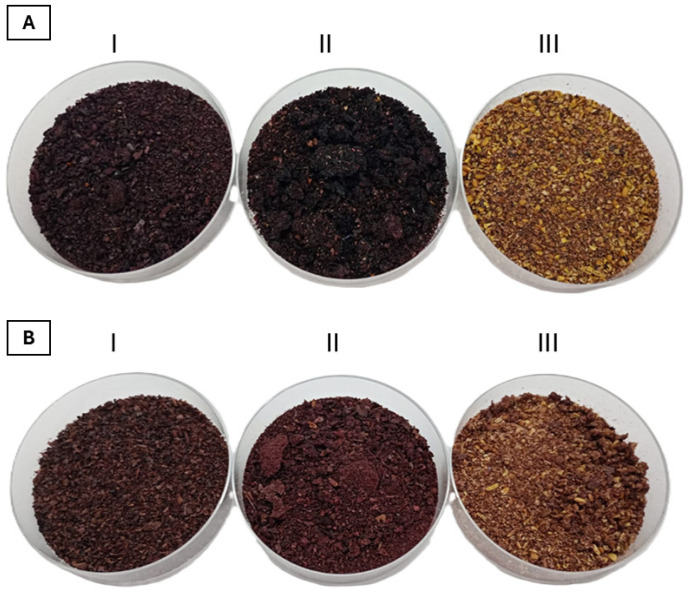
Powders of the peel (**I**), pulp (**II**), and seed (**III**) of (**A**) *Berberis mikuna* and (**B**) *Berberis burruyacuensis* fruits.

Color is one of the first parameters to come into contact with the consumer, so it is one of the most influential factors in consumer food choices, providing an idea of freshness, taste, or composition of food products, which can lead to their acceptability or rejection. This indicator develops and intensifies due to the accumulation of natural pigments (anthocyanins, betalains, carotenoids, alkaloids, flavonoids, and chlorophylls) [17]. The pulp and peel powders of both *Berberis* showed a violet color (Figure 3). The chromatic characteristics of the powders are shown in Table 1. The values of the parameter L* indicate medium luminosity in all fruit parts (L* ≤ 42.07). The values of parameter a* (green to red) were positive (a* values between 2.7 to 8), indicating a preponderance of red over green. The values of parameter b* (blue to yellow) were positive (b* values between 0.07 and 9.58), suggesting the primacy of yellow over blue. The peel and pulp powders of both species show b* values that are lower than those in the seed powders, indicating higher shades of blue. *B. mikuna* showed b* values lower than *B. burruyacuensis*, indicating the highest shades of blue. A natural food colorant based on anthocyanins was demonstrated in other wild barberry (*Berberis vulgaris* L.) fruits [18].

### 2.2. Nutritional and Phytochemical Composition of the Pulp, Peel, and Seed Powders of B. mikuna and B. burruyacuensis

#### 2.2.1. Nutritional Composition of *Berberis* Seed, Pulp, and Peel Powders

The nutritional composition and the moisture content of the peel, pulp, and seed powders of both *Berberis* species are shown in Table 2. To the best of our knowledge, no previous data about these parameters have been published for *B. mikuna* and *B. burruyacuensis*. As expected, the moisture content was higher in the pulp, with no significant differences observed between the two species. The pulp powder of *B. mikuna* fruits showed the highest amount of soluble total sugars (37.55 g GE/100 g of powder). The values are higher than those found in *B. burruyacuensis* pulp or those reported by other authors for native fruits pulp powders, such as chilto [19], and similar to those found in mistol pulp powder [20]. The pulp powder of *B. mikuna* fruits also showed the highest amount of reducing sugars (36.8 g GE/100 g), while its seeds contained the lowest level of reducing sugars. The highest amount of protein was found in the seed powders of both species, with maximum values of 12.8% for *B. mikuna* and 14.9% for *B. burrayucuensis*. A similar protein content was found in chickpea flour [21] and in the seeds of avocado, watermelon, and melon [22]. These values are comparable to those previously reported for *Berberis microphylla* fruits [23] and higher than those reported by Ruiz et al. (2010) [24] for *B. microphylla* fruits, while the protein content in the pulp was similar to that observed in other *Berberis* species, such as *B. asiatica* (pulp: 5.5%; seed: 5.9%), *B. aristata* (pulp: 6.3%; seed: 6.5%), *B. lycium* (pulp: 6.2%; seed: 7.1%), *B. jaeschkeana* (pulp: 4.7%; seed: 6.1%), and *B. pseudumbellata* (pulp: 7.2%; seed: 8.5%) [25].

As in the case of protein, the highest amount of fat was found in the seeds of both species, exhibiting maximum values of 1.5% for *B. mikuna* seeds and 2.4% for *B. burruyacuensis* seeds. The amount of fat found in the pulp and seeds of both *Berberis* species was lower than those of *B. asiatica* (pulp: 3.1%; seed: 5.3%), *B. aristata* (pulp: 3.7%; seed: 5.2%), *B. lycium* (pulp: 2.9%; seed: 4.8%), *B. jaeschkeana* (pulp: 2.6%; seed: 4.7%), and *B. pseudumbellata* (pulp: 4.0%; seed: 4.6%) [25]. The low lipid content of *B. mikuna* and *B. burruyacuensis* seeds was similar to avocado seeds [22].

The total fiber content in all powders of both *Berberis* species was higher than *B. asiatica* pulp (8.1%) and *B. pseudumbellata* seeds (5.2%) [25]. The seed and peel of *B. mikuna* and *B. burruyacuensis* showed the highest levels of fiber, similar to passion fruit seeds [22] (Table 2).

The ash content of foods is often utilized as a measure of mineral levels. The total ash content of *B. burrayucuensis* and *B. mikuna* fruits was higher than reported for calafate (0.64 to 1.3%) [24]. The *B. mikuna* seed powder showed an ash content (1.33 g/100 g) higher than that reported for several fruits (ranging from 0.20 to 0.69 g/100 g in fruits) and similar to minor levels reported in cereals (1.62 to 3.80 g/100 g) [22,26].

The results obtained on the proximal composition of these powders indicate that seeds, which are generally undervalued and considered waste, could be used as an important source of minerals, fiber, and proteins, and that these results can encourage their consumption.

#### 2.2.2. Phytochemical Composition

Wild fruits are known to be rich in various types of phenolic compounds, including flavonoids, anthocyanins, tannins, and phenolic acids. Phenolic compounds play a crucial role in the functional properties of fruits and contribute to the flavor, astringency, and bitterness of fruits and foods [27]. In this study, the content of total phenolic compounds (TPCs), flavonoids (F), condensed tannins (CTs), and anthocyanins in the seed, peel, and pulp powders of two *Berberis* species from NWA were determined for the first time (Table 3). The pulp showed TPC values of 0.78 g GAE/100 g powder (equivalent to 313.34 mg GAE/100 g FW) for *B. burruyacuensis* and 0.76 g GAE/100 g powder (equivalent to 268.31 mg GAE/100 g FW) for *B. mikuna*. Boeri et al. (2020) [23] reported a high phenolic compound content in the pulp of *B. microphylla* (1035.03 mg GAE/100 g FW). Similarly, TPC levels in the pulp of other *Berberis* species have been documented, including *B. vulgaris* (1052.3 mg GAE/100 g FW), *B. amurensis* (923.2 mg GAE/100 g FW), *B. canadians* (899.2 mg GAE/100 g FW), *B. koreana* (1362.8 mg GAE/100 g FW), and *B. declinata* (1243.2 mg GAE/100 g FW) [28]. The TPC content of *B. mikuna* seeds (24.54 g GAE/100 g DW) and *B. burruyacuensis* (29.83 g GAE/100 g DW) were considerably higher than the values reported for grape seeds from various cultivars, which range from 7.90 to 11.12 g GAE/100 g DW [29]. Similarly, the TPCs measured in the pulp of *B. mikuna* (1.48 g GAE/100 g DW) and *B. burruyacuensis* (1.77 g GAE/100 g DW) exceeded those previously reported for blueberry pulp from different varieties, which ranged between 0.15 and 0.69 g GAE/100 g DW [30]. In contrast, the TPC values recorded in the peel of *B. burruyacuensis* (1.61 g GAE/100 g DW) and *B. mikuna* (1.59 g GAE/100 g DW) were lower than those found in the peel of various blueberry cultivars, which showed a broader range from 2.5 to 6.9 g GAE/100 g DW [30]. The flavonoid content determined in different fruit anatomical parts of *B. mikuna* and *B. burruyacuensis* was notably higher than the values reported for grapes (*Vitis vinifera*) from various cultivars. In the seeds, *B. mikuna* and *B. burruyacuensis* presented values of 116.73 and 162.60 g QE/100 g DW, respectively, substantially exceeding the flavonoid content reported for grape seeds (4.00–5.20 g QE/100 g DW) [29]. In the pulp, the flavonoid content was 0.44 g QE/100 g FW in *B. mikuna* and 0.84 g QE/100 g FW in *B. burruyacuensis*, which are considerably higher than the values reported for grape pulp (0.12–0.13 mg QE/100 g FW) [31]. In the peel, *B. mikuna* and *B. burruyacuensis* exhibited values of 2.97 and 4.13 g QE/100 g DW, respectively, which greatly surpassed the flavonoid content reported for grape peels (9.8–40.0 mg QE/100 g DW) [32]. These findings underscore the high flavonoid content of *Berberis* fruits, highlighting their potential as valuable sources of antioxidant bioactive compounds.

The amounts of total phenolic compounds, flavonoids, and condensed tannins in the pulp and peels were similar between both species. The seeds of the fruits of both species had approximately 10 times higher TPC values than the pulp and peel, while flavonoid levels were 20 to 40 times higher than the pulp and peel (Table 3). The same trend was observed for tannins. The tannin levels were higher than those reported for other *Berberis* species, including *B. jaeschkeana* (1.4 mg/100 g), *B. pseudumbellata* (1.5 mg/100 g), *B. aristata* (1.9 mg/100 g), *B. lycium* (2.1 mg/100 g), and *B. asiatica* (2.4 mg/100 g) [27]. Tannins are commonly described as antinutritional compounds; however, they exhibit dual effects on health. On the one hand, they are considered antinutrients because they can reduce mineral absorption, inhibit digestive enzymes, and alter protein bioavailability, which may have a detrimental impact on nutrition, especially in diets high in tannins. Nevertheless, in recent years, tannins have been studied for their numerous biological benefits, including antioxidants, anti-inflammatory, neuroprotective, and anticancer properties. These benefits are mainly observed at moderate doses, demonstrating that tannins can act as functional compounds when consumed in appropriate amounts [33].

Anthocyanins were detected in all parts of both fruits; however, the pulp and peel powders of *B. mikuna* exhibited higher anthocyanin content than those of *B. burruyacuensis*. These findings align with the observed violet coloration of both powders (Table 1 and Figure 3). Similarly, previous studies have reported the incorporation of *B. boliviana* whole-berry powder into yogurt matrices, resulting in an attractive, stable anthocyanin-rich product that eliminates the need for industrial colorants [34]. *B. mikuna* exhibits a higher anthocyanin concentration compared to *B. asiatica*, which has been reported to contain 219 mg C3GE/100 g, highlighting its potential as a superior natural source of these bioactive pigments [35]. Levels of anthocyanins in the peel and pulp of *B. mikuna* and *B. burruyacuensis* were notably higher than the values reported for blueberries (*Vaccinium corymbosum*). In the pulp, *B. burruyacuensis* and *B. mikuna* presented values of 308.73 and 556.76 mg C3G/100 g DW, respectively, exceeding the anthocyanin content reported for blueberry pulp (5.8 mg C3G/100 g DW). Likewise, the anthocyanin content of *B. burruyacuensis* (164.95 mg C3G/100 g DW) and *B. mikuna* (503.80 mg C3G/100 g DW) peels were higher than the anthocyanin content reported for blueberry peel (188.5 mg C3G/100 g DW) [36].

#### 2.2.3. Identification of Phytochemicals Using UHPLC-MS

**Figure 4:**
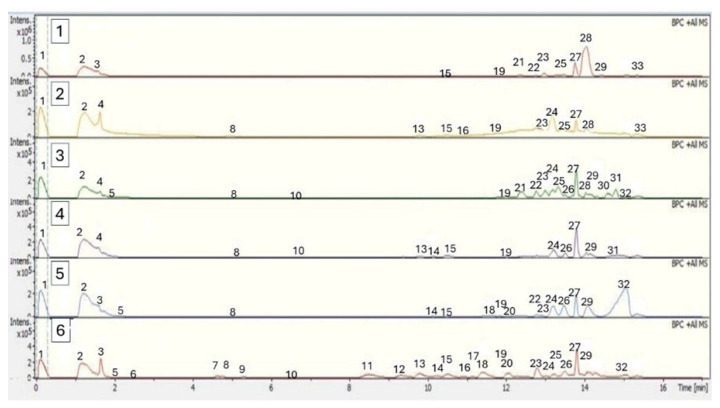
Chromatograms of the basic HPLC-MS peaks in positive mode. (**1**) *B. burruyacuensis* peel; (**2**) *B. mikuna* peel; (**3**) *B. burruyacuensis* pulp; (**4**) *B. mikuna* pulp; (**5**) *B. burruyacuensis* seed; (**6**) *B. mikuna* seed.

To analyze the phytochemical composition of the *Berberis* powders, ethanolic extracts were prepared. Table 4 shows the retention time and MS/MS spectral data of the bioactive compounds present in the pulp, peel, and seed extracts of *B. mikuna* and *B. burruyacuensis* fruits characterized by UHPLC-MS. This study represents the first chemical characterization of these species using this methodology, resulting in the tentative identification of 33 compounds. The base peak chromatograms of *Berberis* extracts were obtained in the positive ion mode, as shown in Figure 4.

The identified metabolites in the pulp, seed, and peel of both *Berberis* species were mainly alkaloids, phenolic compounds (a xanthone, a phenolic ester, coumarins, flavonoids, anthocyanins, and tannins), amino acids, lipids, and vitamins.

Alkaloids

The main alkaloid found in *Berberis* was reticuline (peak 9, C_19_H_23_NO_4_), which is similar to trigonelline (peak 6, C_7_H_7_NO_2_), N-methylcoclaurine (peak 7, C_18_H_21_NO_3_), tetrahydropalmatine (peak 17, C_21_H_25_NO_4_), and alanopine (peak 11, C_6_H_11_NO_4_), which were exclusively detected in the seeds of *B. mikuna*.

Additionally, berberine (peak 10, C_20_H_18_NO_4_) was found in the pulp of both species and in the seeds of *B. mikuna*. Oxyberberine (peak 24, C_20_H_17_NO_5_) was detected in almost all fruit parts, except for the peel of *B. burruyacuensis*. Columbamine (peak 29, C_20_H_20_NO_4_) was found in nearly all fruit parts, except for the peel of *B. mikuna*. Tetrahydrocolumbamine (peak 22, C_20_H_23_NO_4_) was exclusively present in the fruit parts of *B. burruyacuensis*. Allocryptopine (peak 18, C_21_H_23_NO_5_) was detected in the seeds of both species. Some of these compounds, such as berberine, oxyberberine, tetrahydropalmatine, and columbamine, have been previously reported in other *Berberis* species [15,37]. Alkaloids are responsible for various pharmacological activities, such as antioxidants, anti-inflammatory, antiviral, antibacterial, antiparasitic, anticancer, and antiarrhythmic properties, among others. Among other compounds, berberine and its derivatives, such as oxyberberine, have been evaluated and found effective in the prevention and treatment of metabolic syndrome [15,38,39].

Amino acids

Three amino acids were tentatively identified, L-histidine (peak 2, C_6_H_9_N_3_O_2_), L-glutamic acid (peak 3, C_5_H_9_NO_4_), and tryptophan (peak 13, C_11_H_12_N_2_O_2_). L-histidine was found in all parts of the fruit and in both species, while L-glutamic acid was found in the peel of *B. burruyacuensis* and in the seeds of both species. Finally, tryptophan was found in all parts of the fruits of *B. mikuna*. These amino acids have also been reported in other *Berberis* fruits, such as *B. heteropoda* fruits [40]. In addition to their nutritional value, some biological properties, such as antioxidant activities, were attributed to these amino acids [41,42,43].

Phenolic compounds

*Hydroxycinnamic acids*

Chlorogenic acid (peak 16, C_16_H_18_O_9_) was detected in the peel and seeds of *B. mikuna* fruits, while its related compound, caffeic acid (peak 5, C_9_H_8_O_4_), was found in the seeds of both species and in the pulp of *B. burruyacuensis*. These compounds have also been previously identified in other *Berberis* fruits, such as *B. microphylla* (calafate), from four different locations in southern Chile [44]. The main pharmacological effects of these compounds include antioxidant, anti-inflammatory, antiviral, antibacterial, hypoglycemic, lipid-lowering, cardio-protective, anticancer, antimutagenic, and immunomodulatory effects, among others [45,46].

*Coumarins*

5,7-Dihydroxy-4-methylcoumarin (peak 21, C_10_H_8_O_4_) was detected in the peel and pulp of *B. burruyacuensis*, while mellein (peak 33, C_10_H_10_O_3_) was found exclusively in the peels of both species collected in Tucumán. *B. lycium* is a source of several coumarins, including 7-hydroxycoumarin (C_10_H_8_O_3_) and 4-methyl-7-hydroxycoumarin, as well as feselol. Coumarins are known for their potential therapeutic effects, including anti-inflammatory and anticoagulant properties [47].

*Xanthones*

Xanthones are natural polyphenolic compounds found in various plant species, as well as in fungi and lichens. Their significance in the medical and pharmaceutical fields lies in their ability to interact with multiple biological targets, endowing them with antioxidant, anti-inflammatory, and neuroprotective properties. Among their main biological activities, they exhibit anticancer, antimicrobial, anti-inflammatory, and neuroprotective effects. Specifically, they have been shown to inhibit cell proliferation in various cancer cell lines, exert activity against pathogenic microorganisms, modulate COX-2 enzyme activity to reduce inflammatory processes, and block cholinesterases, suggesting a potential application in Alzheimer’s treatment [48]. Moreollic acid (peak 32, C_34_H_41_O_9_), a xanthone, was identified for the first time in *B. mikuna* and *B. burruyacuensis* and was detected in the seeds of both species, as well as in the pulp of *B. burruyacuensis*. As reported by Sukpondma et al. (2005) [49], moreollic acid, derived from *Garcinia hanburyi* fruits, displayed antibacterial activity against methicillin-resistant *Staphylococcus aureus*. Additionally, Jin et al. (2019) [50] demonstrated that moreollic acid also exhibited inhibitory effects on α-glucosidase in *Garcinia hanburyi* fruits.

*Flavonoids*

Quercetin-4-O-glucoside (peak 14, C_21_H_20_O_12_) was detected in the seed extracts of both species and in the pulp of *B. mikuna*. Rutin (peak 15, C_27_H_30_O_16_) was identified in almost all fruit parts of both species, except for the pulp of *B. burruyacuensis*, whereas isorhamnetin-3-O-rutinoside (peak 23, C_28_H_32_O_16_) was present in all fruit parts of both species, except for the pulp of *B. mikuna*. Alpinetin (peak 25, C_16_H_14_O_4_) was identified in the peel and pulp of *B. burruyacuensis* and in the peel and seeds of *B. mikuna*.

Epicatechin (peak 20, C_15_H_14_O_6_) was detected in the seeds of both fruits. This compound was previously reported to occur in *B. vulgaris* [51]. Some biological activities attributed to these chemical compounds are antioxidant, anti-inflammatory, antiviral, antimicrobial, antihypertensive, vasorelaxant, neuroprotective, anticancer, hepatoprotective, cardio-protective, anti-proliferative, and anti-atherosclerotic effects, among others [52,53,54,55,56].

*Isoflavones*

**Figure 5:**
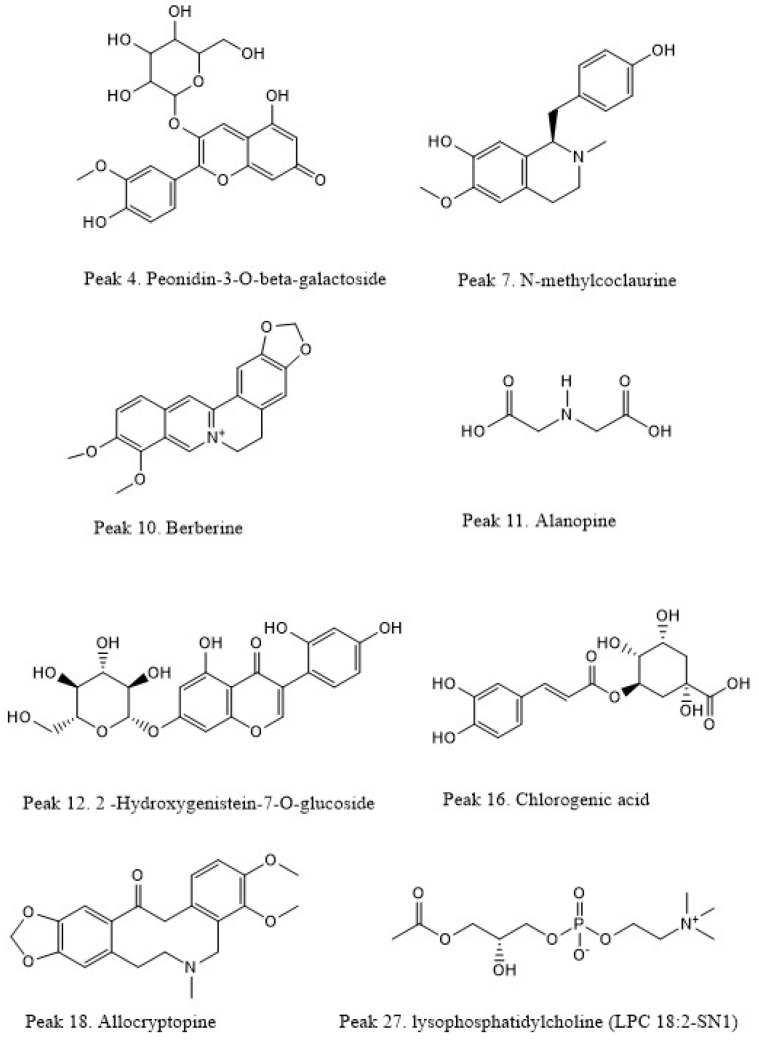
Structures of some representative compounds that were detected in *B. burruyacuensis* and *B. mikuna*.

2-Hydroxygenistein-7-O-glucoside (peak 12, C_21_H_20_O_11_), an isoflavone, was identified for the first time in *B. mikuna* seeds (Table 4, Figure 5).

*Anthocyanins*

Anthocyanins are a class of flavonoids within the polyphenol family that are responsible for the red, purple, and blue colors in many fruits and vegetables.

Peonidin-3-O-β-galactoside (peak 4, C_22_H_22_O_11_) was detected in the pulp of both Berberis species and in the peel of *B. mikuna* (Table 4, Figure 4). This pigment gives cherry red hue shades in plants. It has also been reported that the consumption of anthocyanin-rich foods can reduce the risk of cardiovascular diseases, improve cognitive function, and protect against cancer [56].

*Procyanidins*

Procyanidin B1 (peak 19, C_30_H_26_O_12_), a tannin, was detected in all anatomical parts of the fruit in both species. Tannins are polyphenolic compounds synthesized by higher plants as specialized metabolites for defense. These compounds are widely distributed in plants, particularly in roots, stems, bark, leaves, seeds, and fruits. In foods, tannins contribute to astringency and bitterness and are present in products such as coffee, cocoa, tea, red wine, nuts, and legumes. Additionally, tannins have been extensively studied for their health benefits, including antioxidant, anti-inflammatory, and antimicrobial properties, as well as their potential role in the prevention of cardiovascular diseases and certain types of cancer [57,58].

*Fatty acids*

The analysis of the fruits allowed the identification of various fatty acids, such as erucic acid (peak 30, C_22_H_42_O_2_), which was found exclusively in the pulp of *B. burruyacuensis*. Among the identified compounds, two phosphatidylcholines (PC) were detected: lysophosphatidylcholine (LPC 18:3-SN1) (peak 26, C_26_H_48_NO_7_P), identified in the pulp and seeds of both fruits, and lysophosphatidylcholine (LPC 18:2-SN1) (peak 27, C_26_H_50_NO_7_P), which was detected in all anatomical parts of both fruits. These molecules are essential components of cell membranes, contributing to their structure and functionality. Furthermore, the detection of essential fatty acids, such as linolenic acid (peak 28, C_18_H_30_O_2_) and linoleic acid (peak 31, C_18_H_32_O_2_), is particularly noteworthy.

These essential fatty acids were also reported in other *Berberis* species [59]. Linoleic acid is an essential fatty acid found in foods such as sunflower oil and nuts. It has anti-inflammatory properties and can improve cardiovascular health [60].

*Vitamins*

Ascorbic acid (peak 8, C_6_H_8_O_6_) was identified in all fruit except for the peel of *B. burruyacuensis*. It was also found in other *Berberis* species, such as *B. vulgaris* [61]. Some biological activities attributed to this compound include antioxidant, anti-inflammatory, and anticancer effects [62].

### 2.3. Functional Properties of Berberis burruyacuensis and Berberis mikuna Seed, Pulp, and Skin Powders

The biological activities of extracts obtained from *Berberis* powders were determined.

#### 2.3.1. Antioxidant Activity

Table 5 shows the antioxidant activity values, as measured by ABTS^•+^ and the hydrogen peroxide scavenging values of the different fruit parts (peel, pulp, and seed) of the two *Berberis* species. All the powder extracts showed a dose–response effect with regard to their ABTS cation radical scavenging capacity, with SC_50_ values between 0.71 ± 0.01 and 1.80 ± 0.14 μg GAE/mL. Overall, the scavenging activity was similar to quercetin, a commercial natural antioxidant (SC_50 quercetin_ = 0.85 ± 0.04 μg/mL).

All extracts showed a high H_2_O_2_ scavenging effect (Table 5). The seed extracts of both *Berberis* species were more effective than the other extracts, with SC_50_ values of 24.8 and 32.4 μg GAE/mL. The *B. mikuna* peel extract, with an SC_50_ value of 22.4 µg GAE/mL, was able to scavenge only 37.4% of H_2_O_2_. Although hydrogen peroxide itself is not very harmful, it can generate other highly reactive species, such as HO•, which is responsible for diseases linked to oxidative, infectious, and inflammatory processes [63].

The antioxidant activity of extracts obtained from *B. burruyacuensis* and *B. mikuna* powders could be attributed to the presence of phenolic compounds identified in the present study, such as chlorogenic acid, caffeic acid, isorhamnetin derivatives, moreollic acid, rutin, procyanidin B1, and other compounds (Table 5), which demonstrated antioxidant activity [45,54,55,58]. Alkaloids such as berberine have been reported in various *Berberis* species and could also contribute to the antioxidant effect [15,38].

These results demonstrate that *B. mikuna* and *B. burruyacuensis* powders, or the extracts obtained from them, could be used as functional ingredients to improve the oxidative state.

#### 2.3.2. Effect of *Berberis* Fruit Powder Extracts on Enzymes Involved in the Development of Metabolic Syndrome

Recent reports suggest that the development of metabolic syndrome is associated with various clinical health disorders, including oxidative stress, proinflammatory states, and non-alcoholic fatty liver disease [64]. Metabolic syndrome is characterized by the development of hypertension, hyperglycemia, and hypercholesterolemia [65]. Previous reports suggest that bioactive compounds present in so-called “red fruits”, particularly polyphenols such as phenolic acids and anthocyanins, may have beneficial effects against the development of obesity and other diseases [66]. In this context, the activity of extracts from *B. mikuna* and *B. burruyacuensis* fruit powders against key enzymes involved in metabolic syndrome was evaluated, and the results are presented in Table 6.

The search for natural products capable of inhibiting pancreatic α-amylase and α-glucosidase activity represents a therapeutic strategy for the control of postprandial hyperglycemia.

All extracts obtained from *Berberis* fruit powders actively inhibited both enzymes, with IC_50_ values between 2.1 and 51.6 µg GAE/mL for α-glucosidase and 3.5 and 33.6 µg GAE/mL for α-amylase (Table 6). The powdered seed extracts exhibited the highest inhibitory activity against both enzymes. This potency may be due to the greater diversity of polyphenols and alkaloids found in this study in seed powders (Table 4). The polyphenols present in these powders, such as caffeic acid, quercetin, rutin, procyanidin, isorhamnetin, and xanthones, could be responsible for this effect. Polyphenols can reach the intestine, even when diluted with other foods and digestive fluids, where they interact with digestive enzymes and modulate glycemic responses by inhibiting carbohydrate digestion [67]. Several alkaloids, such as berberine and oxyberberine, had also demonstrated inhibitory activity against these enzymes and were found in the seed powder of both plant species [15,38,39]. It is important to note that the seeds, in addition to presenting a greater diversity of phenolic compounds (Table 4), have the highest content of total phenolic compounds, flavonoids, and condensed tannins (Table 3).

The pulp and peel extracts were also active against both enzymes, although with less potency. The weaker inhibitory effect is likely due to the lower polyphenol diversity (Table 4). The anthocyanins contained in the pulp of *B. mikuna* and *B. burruyacuensis* (Table 3 and Table 4) probably influence the activity of these enzymes. Previous work indicated the inhibitory effect of anthocyanin isolates from other *Berberis* fruits on α-glucosidase and α-amylase [68]. Furthermore, in vivo studies report that anthocyanins present in fruits improve insulin sensitivity through complex biochemical mechanisms, thus potentially preventing diabetes. Animal models of diabetes, as well as cross-sectional studies in humans, revealed that anthocyanins reduce blood glucose levels and peripheral insulin resistance [69,70].

Pancreatic lipase breaks down triglycerides into absorbable glycerol and fatty acids. Its inhibition by drugs such as orlistat has been used to treat obesity, with numerous undesirable side effects. However, several studies have shown that polyphenols present in fruits have lipase-inhibiting effects and could constitute a healthy way to regulate fat digestion, and therefore energy intake and obesity [71]. All extracts obtained from Argentine *Berberis* fruit powders were potent inhibitors of pancreatic lipase, with IC_50_ values between 0.26 and 1.16 µg GAE/mL, particularly the seed extracts (Table 6). Sosnowska et al. (2022) [72] indicated that extracts obtained from chokeberry fruits rich in proanthocyanidins were potent inhibitors of the lipase enzyme, while those fractions containing mainly low-molecular-weight phenolic compounds, such as phenolic acids, flavonols, and anthocyanins, were less active. In this framework, we can conclude that the seed extracts of both *Berberis* were more active in inhibiting pancreatic lipase due to the presence of compounds such as condensed tannins in their composition. Polyphenol-rich extracts from *Berberis* fruits showed greater potency in inhibiting pancreatic lipase than extracts from *Solanum betaceum* fruits, both red and orange varieties, as well as from beverages made with *Zuccagnia punctata* extracts, blueberry juice, and lemon honey [19,73,74].

### 2.4. Toxicity Analysis of Berberis burruyacuensis and Berberis mikuna Seed, Pulp, and Skin Powders

The results obtained from the *A. salina* assay showed that *B. mikuna* and *B. burruyacuensis* powder extracts were not toxic below a concentration of 125 μg GAE/mL. Table 7 shows the results obtained in the assay evaluating mutagenic activity against *Salmonella* Typhimurium strains TA98 and TA100. The mutagenicity assay using *S.* Typhimurium strains TA98 and TA100 indicated that, up to a concentration of 500 μg GAE/plate, *B. mikuna* and *B. burruyacuensis* extracts did not induce an increase in the number of spontaneous revertants, showing, in all cases, an RM < 1.5. It can be concluded that the extracts evaluated are neither toxic nor genotoxic at the concentrations tested, which would guarantee their safe use.

## 3. Materials and Methods

### 3.1. Chemicals and Reagents

Ethanol, phenol, sulfuric acid, and H_2_O_2_ were purchased from Cicarelli (Santa Fe, Argentina). Folin–Ciocalteau reagent, AlCl_3_, gallic acid, quercetin, procyanidin B2, and ABTS^•+^ were acquired at Sigma Aldrich, St. Louis, MO, USA. α glucosidase, α amylase, lipase, p-nitrophenyl-α-D-glucopyranoside, p-nitrophenyl palmitate, and acarbose were from Sigma-Aldrich (Darmstadt, Germany). Amilokit ^®^ was purchased from Wiener Lab Group, Rosario, Argentina, Kit No. 1504163370. Orlistat (tetrahydrolipstatin, ATC code: A08AB01) was purchased from Elea Laboratory, Ciudad Autónoma de Buenos Aires, Argentina.

### 3.2. Plant Material

*B. mikuna* fruits were collected in January at the coordinates 26°23′52.8″ S 65°04′22.2″ W and *B. burruyacuensis* fruits were collected in February at the coordinates 26°23′52.8″ S 65°04′20.3″ W (Figure 2). Voucher specimens were deposited at the Herbarium of Fundación Miguel Lillo, Tucumán, Argentina (LIL 618421 and 618422, respectively). The identification of plant materials was carried out by the botanist Dr. Ana Soledad Cuello. The harvesting stage of fruits was selected as ripe grade as commonly consumed (Figure 1). Once transferred to the laboratory, the fruits of both species were washed with tap water and separated into peel, pulp, and seed. Subsequently, each part of the fruit was dried by freeze-drying and then ground in a Helix mill (Numak, F100 Power 1/2 HP-0.75 Kw, Brusque, Brazil) to obtain a fine powder (Figure 3). The powders were stored in sealed plastic bags at −20 °C.

### 3.3. Quality Parameters of Fruits

The chromatic parameters of powders from each part of the fruit (pulp, peel, and seed) were measured with a Chroma meter NR110 (3NH TECHNOLOGY Co., Ltd., Zengcheng, China) using the CIELab system. The color space was chosen to obtain the results expressed in the chromaticity coordinates L*, a*, and b* for the selected illuminant. The L* coordinate represents the lightness (black or white contribution varying between 0 and 100), a* represents the green or red contribution (negative or positive), and b* represents the blue or yellow contribution (negative or positive). The L* coordinate is perpendicular to the plane containing the chromaticity coordinates a* and b*. Considering the L*, a*, and b* coordinates, the color is expressed through L*, C*, and H, where L* is the brightness, C* is the chroma or saturation, and H is the hue (or hue angle, indicating the color variation in the plane formed by the a* and b* coordinates). These parameters were determined considering: C* = (a2 + b2) 1/2, H° = arc tang (b/a), where 0° = red-purple, 90° = yellow, 180° = bluish-green, and 270° = blue and the CIRG index= 180-h/(L* + C*) [75].

### 3.4. Nutritional Analysis

To 0.2 g of each one of the powders, 3 mL of 80% ethanol was added and heated at 80 °C for 10 min. The extractions were carried out until exhaustion. Then, they were centrifuged and the total soluble sugars in the supernatant were quantified using the method proposed by Dubois et al. (1956) [76]. The results were expressed in g of glucose equivalents in 100 g of powder (g GE/100 g of powder). Reducing sugars were quantified by Somogyi (1945) [77] and Nelson (1944) [78]. The results were expressed in g of glucose equivalents in 100 g of powder (g GE/100g of powder). Total protein content was determined by measuring total nitrogen (N) using the Kjeldahl method with a conversion factor of 6.25 (AOCS, 1989) [79]. The total lipid content in each of the powders was determined gravimetrically after continuous extraction of crude fat with petroleum ether at 40–60 °C in a Soxhlet for 4 h. The results were expressed in g/100 g powder.

The moisture content was determined by evaluating the difference in weight between the fresh sample and the sample dried by freeze-drying. The dried sample was placed in a muffle furnace (500 °C) until the ashes were obtained (AOAC, 2005) [80]. The results were expressed in g/100 g powder.

Crude fiber content was determined according to AOAC (2005) [80]. The powder (0.4 g) was weighed and transferred to a beaker containing 4 mL of 1.25% H_2_SO_4_. The mixture was incubated at 100 °C for 30 min. Subsequently, 10 mL of NaOH 3.52% was added, and the mixture was incubated again at 100 °C for 30 min. The sample was filtered and subsequently washed with hot distilled water until the alkali was eliminated. It was then washed with H_2_SO_4_ 1.25% and again with distilled water until the acid was eliminated. Finally, the sample was placed on ash-free filter paper in a crucible. The sample was transferred to ash-free filter paper in a pre-weighed porcelain crucible and dried at 110 °C until a constant weight. The sample was then incinerated in a muffle furnace at 500 °C, and the crude fiber content was determined by difference, with the result expressed in g/100 g powder.

### 3.5. Functional Phytochemicals

#### 3.5.1. Extract Preparation

The ethanolic extractions were performed using 1 g of each part of the fruit (seeds, pulp, and peel) powders with 10 mL of 96° ethanol, assisted by ultrasound in 3 cycles of 20 min each. The extracts were then filtered and stored in caramel-colored glass bottles. The dry weight (DW) of each extract was determined by evaporating the solvent at a constant temperature of 40 °C. Dry extracts were stored frozen at −20 °C until analysis.

#### 3.5.2. Total Polyphenols and Flavonoids

The content of total phenolic compounds was quantified using the technique described by Singleton et al. (1999) [81]. Each extract was mixed with 0.1 mL of Folin–Ciocalteu reagent (previously diluted with distilled water, 1:1; *v*:*v*) and 0.4 mL of 15.9% sodium carbonate solution. The mixture was then incubated at room temperature for 20 min. After incubation, the absorbance was measured at 765 nm using a UV–visible spectrophotometer (UV-2400 PC). The results were expressed in g of gallic acid equivalents (GAE) in 100 g of powder (g GAE/100 g powder). The flavonoid content was estimated using the method of Zhishen et al. (1999) [82]. Each extract was mixed with 0.03 mL of 5% sodium nitrite solution and allowed to stand for 5 min. Subsequently, 0.03 mL of 10% aluminum chloride solution was added, followed by a 5 min incubation period. Then, 0.2 mL of 1% sodium hydroxide solution was added. The reaction mixture was brought to the final volume with distilled water. Absorbance was then measured at 510 nm using a UV–visible spectrophotometer (UV-2400 PC). The results were expressed in g of quercetin equivalents in 100 g of powder (g QE/100 g powder).

#### 3.5.3. Condensed Tannins and Pigments

The seed, pulp, and peel extracts were used to determine the condensed tannin and anthocyanin contents. The total condensed tannin content was quantified as described by Prior et al. (2010) [83]. The condensed tannin content was determined by mixing 30 μL of each sample with 900 μL of 0.1% DMAC (4-Dimethylaminocinnamaldehyde) in acidified ethanol and 270 μL of 80% ethanol. The mixture was incubated for 20 min at 25 °C. Absorbance was then measured at 640 nm. Results were expressed as g of procyanidin B2 equivalent (PB2E) in 100 g powder (g PB2E/100g powder) of each fruit part.

The anthocyanin content was quantified using the differential pH method according to the technique described by Lee et al. (2005) [84]. This spectrophotometric method is based on the structural transformation of anthocyanins with a change in pH (pH 1.0—colored and pH 4.5—uncolored). The samples were diluted in a solution of potassium chloride (0.025 M pH 1.0) and sodium acetate buffer (0.4 M pH 4.5) in a 4:1 ratio (buffer/sample). The solution was incubated for 20 min in the dark, and the absorbance readings were taken at 520 and 700 nm. The total anthocyanin concentration was calculated using the following formula:

Total anthocyanins (mg/L)= (A × MW × DF × 10^3^)/ɛ × l)

where A (absorbance) = (A_520nm_ − Abs_700nm_)_pH1_ − (A_520nm_ − A_700nm_)_pH4.5_; MW = the molecular weight of cianidin-3-glucoside: 449.2 g/mol; DF = the dilution factor; ɛ = 26,900; the molar extinction coefficient for cyanidin-3-glucoside (L/mol/cm); l = the path length (cm). The results were expressed in mg of cyanidin-3-glucoside equivalents in 100 g of powder (mg C3GE/100 g of powder).

#### 3.5.4. Identification of Phytochemicals Using UHPLC-PDA-ESI-QT-MS/MS

The separation and identification of specialized metabolites from *B. mikuna* and *B. burruyacuensis* extracts were carried out on a UHPLC-MS system equipped with a UHPLC Ultimate 3000 RS with Metaboscape 4.0 software, and a Bruker maXis ESI-QTOF-MS. The chromatographic equipment consisted of a quaternary pump, an autosampler, a thermostated column compartment, and a photodiode array detector. The elution was performed using a binary gradient system with eluent (A), 0.1% formic acid in water, and eluent (B), 0.1% formic acid in acetonitrile, and the following gradient: isocratic 12% B (0–1 min), 12–99% B (1–11 min), isocratic 99–99% B (11–13.5 min), 99–12% B (13.5–14 min), and 12–12% B (14–15 min). The separation was carried out with an acclaim Kinetex C18 1.7 μm (2.1 mm × 100 mm) column at a flow rate of 400 mL/min. ESI-QTOF-MS experiments in negative ion mode were recorded, and the scanning range was between 50 and 2000 *m*/*z*.

### 3.6. Antioxidant Activity of Polyphenolic Extracts

#### 3.6.1. ABTS^●+^ Scavenging Assay

The total antioxidant activity of each extract was measured using the enhanced ABTS radical cation (ABTS^●+^) method, as described by Re et al. (1999) [85]. Results were expressed as the concentration of phenolic compounds in gallic acid equivalent per mL (µg GAE/mL) required to remove 50% of ABTS (SC_50_). Quercetin was used as a reference compound.

#### 3.6.2. Hydrogen Peroxide Scavenging Assay

The ability of polyphenolic extracts from *Berberis* fruits to scavenge hydrogen peroxide was measured according to Fernando and Soysa (2015) [86] with some modifications. Briefly, the reaction mixture containing the polyphenol-enriched extracts (7–70.4 µg GAE/mL), H_2_O_2_ (0.7 mM), and horseradish peroxidase (1 U/mL) was preincubated for 3 min at 37 °C. The mixture containing the phenolic extract (2–50 µg GAE/mL) and H_2_O_2_ (0.7 mM) was incubated for 3 min at 37 °C. Then, a solution of phenol (12 mM) and 4-aminoantipyrine (0.5 mM) was added to the mixture. The absorbance was measured at 504 nm (Spectrophotometer Jasco v-630, Thermo Fisher Scientific, Tokyo, Japan) by the production of a colored quinone. The values were expressed as the concentration of phenolic compounds in gallic acid equivalent per mL (µg GAE/mL) required to remove 50% of hydrogen peroxide (SC_50_). Quercetin was used as a control.

### 3.7. Antihyperglycemic and Antihyperlipidemic Activity of Berberis Extracts

#### 3.7.1. α-Glucosidase Inhibition

The inhibitory potential of the extracts on the glucosidase enzyme was performed according to Costamagna et al. (2013) [87] with some modifications. First, preincubation of the α-glucosidase enzyme (0.0273U) and the polyphenolic extracts of the seeds, peel, and pulp (0.5–70 µg GAE/mL) was performed in 160 µL of 0.1 M sodium phosphate buffer (pH 6.9) at 4 °C for 15 min. Then, 5 μL of 25 mM *p*-nitrophenyl α-D-glucopyranoside was added to start the reaction. After 15 min of incubation at 37 °C, 80 μL Na_2_CO_3_ 0.2 M was added to stop the reaction and the absorbance at 405 nm was recorded. The IC_50_ values were calculated by the interpolation of dose–response curves. Results were expressed as the concentration of phenolic compounds in gallic acid equivalent per mL (µg GAE/mL) required to inhibit 50% of enzymatic activity. Acarbose was used as a positive control.

#### 3.7.2. α-Amylase Inhibition Assay

The inhibitory activity of α-amylase using starch as a substrate was assayed using an Amilokit^®^ according to the manufacturer’s instructions, as reported by Costamagna et al. (2016) [88]. The inhibitory capacity of polyphenolic extracts from the seeds, peel, and pulp (0.5–50 µg GAE/mL) was evaluated and the results are reported as IC_50_ values. The IC_50_ values indicate the µg GAE/mL of extract required to inhibit the enzyme by 50%. Acarbose was used as a positive control.

#### 3.7.3. Lipase Inhibition Assay

The lipase inhibitory activity of *Berberis* extracts was assayed by measuring the enzymatic hydrolysis of *p*-nitrophenyl palmitate to *p*-nitrophenol according to Costamagna et al. (2016) [88]. The enzyme lipase (1 mg/mL) was preincubated with different concentrations of the extracts (final concentration between 0.1 and 4 µg GAE/mL) for 10 min. Then, sodium phosphate buffer 0.1M pH 7 supplemented with 0.6% (*w*/*v*) Triton X-100, 0.15% (*w*/*v*) gum arabic, and 20 µL of 10 mM p-nitrophenyl palmitate (substrate) was added, and the reaction was incubated at 37 °C for 20 min. The absorbance was measured in a microplate reader at 400 nm using a BiotekELx808 microplate reader. IC_50_ values were determined as µg GAE/mL of extracts required to inhibit the enzymatic activity by 50%. Orlistat was used as a positive control.

### 3.8. Toxicity Assessment

#### 3.8.1. Acute Toxicity Assay

The acute toxicity level of extracts from different parts of *Berberis* fruits was evaluated using the crustacean *Artemia salina* as a test organism [89]. *Artemia salina* cysts were incubated in artificial seawater. After 24 h incubation at 25 °C, nauplii were transferred to microplates containing seawater and 15.6–250 µg GAE/mL of each extract. A solvent control (dimethyl sulfoxide, DMSO) without extract and a positive control of potassium dichromate (10–40 µg/mL) were included in the experiment. All plates were incubated for 24 h at 25 °C. At the end of the incubation period, dead larvae were counted for each polyphenolic extract concentration tested.

#### 3.8.2. Mutagenicity

The mutagenic effect of *Berberis* extracts on two strains of *Salmonella* Typhimurium (TA98 and TA100) was evaluated. The plate incorporation assay was performed according to Maron and Ames (1983) [90], adding 0.1 mL of the overnight bacterial culture, 0.1 mL of each extract at different concentrations (125–500 µg GAE/plate), and 2 mL of top agar to minimal agar plates. The plates were then incubated at 37 °C for 48 h. After incubation, revertant colonies were counted and compared with the number of revertant colonies in the controls. The positive control used was 4-nitro-*O*-phenylenediamine (4-NPD; Aldrich Chemical Co., St. Louis, MO, USA), 10 µg/plate. Solvent control was performed by adding 0.1 mL of DMSO/plate. An extract was considered mutagenic when the mean number of revertant was two-fold or more than two-fold higher than that of the negative control. Three plates were tested per experiment and two separate experiments were carried out for each concentration tested and for the positive and negative controls.

### 3.9. Statistical Analysis

The statistical analysis was performed using Infostat software (Student Version, 2015) [91]. All measurements were carried out in triplicate or more, and the data are expressed as mean values accompanied by their respective standard deviations. To evaluate differences among experimental groups, a one-way analysis of variance (ANOVA) was conducted. When significant differences were detected (*p* < 0.05), Tukey’s post hoc test was used to identify specific group differences. A *p*-value below 0.05 was considered indicative of statistical significance.

## 4. Conclusions

This study highlights that fruit powders from *B. mikuna* and *B. burruyacuensis,* two plant species native to Tucumán, Argentina, are promising sources of nutrients (proteins, lipids, and sugars), fiber, and minerals. The nutritional attributes varied considerably among parts of fruits and between species. The powders obtained from seeds, peel, and pulp can also be considered valuable sources of functional compounds, with potential applications as dietary supplements or food additives aimed at preventing diseases associated with oxidative stress and metabolic syndrome. In addition, it is possible to achieve comprehensive use of the fruits using the seeds, which are usually discarded, since it has been shown that they have biological activities such as antioxidant and hypolipidemic effects, which can be attributed to the identified bioactive compounds. The metabolites detected in the pulp, seed, and peel powders of both *Berberis* species were mainly alkaloids and phenolic compounds, including phenolic acids, coumarins, flavonoids, isoflavonoids, xanthones, anthocyanins, and condensed tannins.

Overall, this work provides a foundation for promoting the cultivation and sustainable use of *Berberis* species in NWA.

## Figures and Tables

**Table 1:** CIELAB parameters of the color of *Berberis* seed, pulp, and peel powders.

Plant Species	Fruit Parts	Chromatic Parameters		
		L*	a*	b*
*Berberis burruyacuensis*	Peel	36.14 ± 0.13 ^B^	4.75 ± 0.19 ^A^	1.86 ± 0.15 ^B^
	Pulp	34.58 ± 0.45 ^B^	4.26 ± 0.15 ^B^	0.98 ± 0.09 ^B^
	Seed	42.07 ± 0.40 ^B^	8.00 ± 0.11 ^B^	9.58 ± 0.01 ^B^
*Berberis mikuna*	Peel	34.08 ± 0.01 ^A^	4.15 ± 0.04 ^A^	0.72 ± 0.02 ^A^
	Pulp	33.23 ± 0.12 ^A^	2.70 ± 0.07 ^A^	0.07 ± 0.01 ^A^
	Seed	39.91 ± 0.06 ^A^	4.69 ± 0.01 ^A^	7.42 ± 0.28 ^A^

Values are expressed as mean ± standard deviation (n = 3). Different letters within the same column indicate statistically significant differences in wavelength measurements between fruit parts of both *Berberis* species, as determined by Tukey’s multiple comparison test (*p* < 0.05).

**Table 2:** Nutritional composition of *Berberis* seed, pulp, and peel powders.

Plant Species	Fruit Parts	Moisture ^#^ (%)	Total Soluble Sugars (g GE/100 g Powder)	Reducing Sugars (g GE/100 g Powder)	Total Protein (%)	Fat (%)	Total Fibers (g/100 g Powder)	Ashes (%)
*Berberis burruyacuensis*	Peel	62 ± 3 ^C^	25.38 ± 2.43 ^B^	23.75 ± 2.02 ^B,C^	6.2 ± 0.3 ^A^	2.0 ± 0.1 ^C^	54.04 ± 0.51 ^D^	0.38 ± 0.03 ^A,B^
	Pulp	82 ± 2 ^D^	24.68 ± 1.27 ^B^	26.31 ± 0.05 ^C^	6.9 ± 0.3 ^A^	1.8 ± 0.1 ^C^	25.84 ± 0.48 ^B^	0.41 ± 0.04 ^A,B^
	Seed	47 ± 2 ^B^	26.99 ± 1.11 ^B^	12.07 ± 0.70 ^A^	14.9 ± 0.8 ^C^	2.4 ± 0.1 ^D^	63.86 ± 2.03 ^E^	0.96 ± 0.08 ^C^
*Berberis mikuna*	Peel	57 ± 2 ^C^	23.68 ± 1.40 ^B^	16.03 ± 2.66 ^A,B^	6.1 ± 0.3 ^A^	1.3 ± 0.1 ^B^	35.40 ± 0.53 ^C^	0.50 ± 0.05 ^B^
	Pulp	82 ± 2 ^D^	37.55 ± 1.37 ^C^	36.80 ± 3.96 ^D^	6.3 ± 0.2 ^A^	0.9 ± 0.04 ^A^	12.55 ± 0.85 ^A^	0.26 ± 0.02 ^A^
	Seed	38 ± 2 ^A^	16.72 ± 0.95 ^A^	8.90 ± 0.33 ^A^	12.8 ± 0.7 ^B^	1.5 ± 0.1 ^B^	54.96 ± 2.14 ^D^	1.33 ± 0.11 ^D^

^#^ Moisture of each part of the fresh fruit (pulp, seed, and peel). GE: glucose equivalent. Values are expressed as mean ± standard deviation from triplicate measurements. Different letters within the same column indicate statistically significant differences in the content of nutritional components among all samples, as determined by Tukey’s multiple comparison test (*p* < 0.05).

**Table 3:** Phytochemical content of the seed, pulp, and skin powders of *Berberis burruyacuensis* and *Berberis mikuna*.

Plant Species	Fruit Parts	Total Phenolic Compounds (g GAE/100 g Powder)	Total Flavonoids (g QE/100 g Powder)	Total Anthocyanins (mg C3GE/100 g Powder)	Condensed Tannins (mg PB2/100 g Powder)
*Berberis burruyacuensis*	Peel	0.52 ± 0.01 ^A^	1.35 ± 0.02 ^A^	54.60 ± 0.97 ^B^	0.03 ± 0.00 ^A^
	Pulp	0.78 ± 0.00 ^B^	2.10 ± 0.01 ^A^	137.78 ± 3.18 ^C^	0.04 ± 0.00 ^A^
	Seed	7.94 ± 0.07 ^D^	43.31 ± 0.41 ^C^	20.56 ± 0.65 ^A^	6.09 ± 0.06 ^C^
*Berberis mikuna*	Peel	0.62 ± 0.01 ^A,B^	1.16 ± 0.01 ^A^	195.55 ± 7.75 ^D^	0.06 ± 0.00 ^A^
	Pulp	0.76 ± 0.01 ^B^	1.25 ± 0.02 ^A^	283.49 ± 6.55 ^E^	0.05 ± 0.00 ^A^
	Seed	6.16 ± 0.19 ^C^	29.30 ± 0.93 ^B^	7.82 ± 0.79 ^A^	3.64 ± 0.11 ^B^

Values are expressed as mean ± standard deviation from triplicate measurements. Different letters within the same column indicate statistically significant differences in the content of phytochemical components among all samples, according to Tukey’s multiple comparison test (*p* < 0.05). GAE: gallic acid equivalent; QE: quercetin equivalent; C3GE: cyanidin 3 glucoside; PB2: procyanidin B2 equivalent.

**Table 4:** HPLC-QTOF-MS-Phytochemical identification. (a) Peel, (b) pulp, and (c) seed extracts.

Peak	Tentative Identification	Retention Time (min)	[M + H]^+^	Theoretical Mass (*m*/*z*)	Measured Mass (*m*/*z*)	Accuracy (ppm)	Metabolite Type	MS Ions (ppm)	*B. burruyacuensis* Samples	*B. mikuna* Samples
1	Na formiate (internal standard)	0.37	NaC_2_H_2_O_4_	113.9829	113.9842	2.3	Standard	-	a,b,c	a,b,c
2	L-Histidine	0.6	C_6_H_9_N_3_O_2_	156.0766	156.0733	−21.14	Amino acid	83.0599	a,b,c	a,b,c
3	L-Glutamic acid	0.8	C_5_H_9_NO_4_	148.0609	148.0535	−49.8	Amino acid	84.0456	a,c	c
4	Peonidin-3-O-beta-galactoside	0.9	C_22_H_22_O_11_	463.1211	463.1138	−15.7	Anthocyanin	298.0452, 283.0243, 212.0443	b	a,b
5	Caffeic acid	2.1	C_9_H_9_O_4_	181.0641	181.0622	−21.14	Phenolic acid	83.0599	b,c	c
6	Trigonelline	2.25	C_7_H_7_NO_2_	138.0551	138.0578	19.5	Alkaloid (piridin)	110.0601, 94.0655	-	c
7	N-methylcoclaurine	5.57	C_18_H_21_NO_3_	200.1590	200.1532	−28.9	Alkaloid	269.1181, 107.0491	-	c
8	Ascorbic acid	9.07	C_6_H_8_O_6_	177.0543	177.0566	5.2	Vitamin	115.0547	b,c	a,b,c
9	Reticuline	8.28	C_19_H_23_NO_4_	330.1698	330.1672	−7.8	Alkaloid	192.1023, 177.082	-	c
10	Berberine	8.28	C_20_H_18_NO_4_	336.1446	336.1482	4.7	Alkaloid	320.1128, 292.1154	b	b,c
11	Alanopine2-(1-carboxyethylamino)	9.10	C_6_H_11_NO_4_	162.0764	162.0791	16.6	Alkaloid	116.0692	-	c
12	2-Hydroxygenistein-7-O-glucoside	9.30	C_21_H_20_O_11_	449.1068	449.0996	−16.0	Isoflavone	287.0524	-	c
13	Tryptophan	9.92	C_11_H_12_N_2_O	205.0971	205.0898	−35.5	Amino acid	118.0642, 115.0532	-	a,b,c
14	Quercetin 4-O-glucoside	10.17	C_21_H_20_O_12_	465.1041	465.0969	−15.4	Flavonoid	303.0523, 229.0586	c	b,c
15	Rutin	10.38	C_27_H_30_O_16_	611.1635	611.1662	4.4	Flavonoid	303.0498, 127.0379	a,c	a,b,c
16	Chlorogenic acid	10.69	C_16_H_18_O_9_	355.1014	355.0941	−20.5	Phenolic ester	163.0389	-	a,c
17	Tetrahydropalmatine	11.20	C_21_H_25_NO_4_	356.1789	356.1716	−20.4	Alkaloid	176.0778, 148.0768	-	c
18	Allocryptopine	11.25	C_21_H_23_NO_5_	370.1650	370.1677	7.29	Alkaloid	188.0707, 149.0596	c	c
19	Procyanidin B1	11.40	C_30_H_26_O_12_	579.1488	579.1535	8.11	Tannin	407.0802, 290.0697	a,b,c	a,b,c
20	Epicatechin	12.11	C_15_H_14_O_6_	291.0853	291.0880	9.27	Tannin	147.0472, 123.0462	c	c
21	5,7-Dihydroxy-4-methylcoumarin	12.81	C_10_H_8_O_4_	215.0162	215.0184	10.23	Coumarin	147.0442	a,b	-
22	Tetrahydrocolumbamine	12.69	C_20_H_23_NO_4_	341.1695	341.1891	57.2	Alkaloid	303.3251, 294.1150	a,b,c	-
23	Isorhamnetin-3-O-rutinoside	12.90	C_28_H_32_O_16_	625.1750	625.1677	−11.6	Flavonoid	271.0239, 243.0265	a,b,c	a,c
24	Oxyberberine	13.38	C_20_H_17_NO_5_	352.1176	352.1165	−3.12	Alkaloid	374.0992 (M + Na), 344.0573,	b,c	a,b,c
25	Alpinetin	13.52	C_16_H_14_O_4_	271.0960	271.0887		Flavonoid	167.0365, 152.0123	a,b	a,c
26	lysophosphatidylcholine (LPC 18:3-SN1)	13.66	C_26_H_48_NO_7_P	518.3227	518.3254	5.20	Lipid	494.3489, 475.3257, 466.3167, 459.3174	b,c	b,c
27	lysophosphatidylcholine (LPC 18:2-SN1)	13.77	C_26_H_50_NO_7_P	520.3380	520.3357	−4.4	Lipid	494.3489, 475.3257, 466.3167, 459.3174	a,b,c	a,b,c
28	Linolenic acid	14.01	C_18_H_30_O_2_	279.2315	279.2242	−26.14	Lipid	95.0875, 67.0538	a,b	a
29	Columbamine	14.15	C_20_H_20_NO_4_	338.3653	338.3623	−8.8	Alkaloid	322.1085, 303.3255, 294.1153	a,b,c	b,c
30	Erucic acid	14.52	C_22_H_42_O_2_	339.3106	339.3258	24.6	Fatty acid	255.2514	b	-
31	Linoleic acid	14.86	C_18_H_32_O_2_	281.2470	281.2487		Fatty acid	255.2432	b	b
32	Moreollic Acid	15.08	C_34_H_41_O_9_	593.3158	593.2945	−29	Xanthone		b,c	c
33	Mellein	13.27	C_10_H_10_O_3_	179.0701	179.07356	19.15	Coumarin		a	a

**Table 5:** Antioxidant activity of extracts from *Berberis burruyacuensis* and *Berberis mikuna* seed, pulp, and skin powders.

Plant Species	Fruit Parts	ABTS^•+^	H_2_O_2_
		SC_50_ (μg GAE/mL)	
*Berberis burruyacuensis*	Peel	1.80 ± 0.14 ^E^	41.5 ± 0.7 ^C^
	Pulp	1.13 ± 0.01 ^C,D^	31.5 ± 0.7 ^B^
	Seed	1.00 ± 0.03 ^B,C^	24.8 ± 0.3 ^A^
*Berberis mikuna*	Peel	1.34 ± 0.00 ^D^	* 22.4 ± 0.4
	Pulp	1.63 ± 0.04 ^E^	52.9 ± 0.5 ^D^
	Seed	0.71 ± 0.01 ^A^	32.4 ± 0.3 ^B^

Values are expressed as means ± standard deviation from triplicate measurements. Different letters within the same column indicate statistically significant differences in antioxidant activity among all samples, according to Tukey’s multiple comparison tests (*p* < 0.05). SC_50_: concentration of extract necessary to scavenge 50% of ABTS, H_2_O_2_; GAE: gallic acid equivalent. * Concentration of extract necessary to scavenge 37.4% of H_2_O_2._

**Table 6:** Antihyperglycemic and antihyperlipidemic activity of seed, pulp, and peel extracts of *B. burruyacuensis* and *B. mikuna*.

Plant Species	Fruit Parts	α-Glucosidase	α-Amylase	Lipase
		IC_50_ (µg GAE/mL)		
*Berberis burruyacuensis*	Peel	26.3 ± 4.8 ^B^	15.9 ± 1.1 ^C^	0.85 ± 0.01 ^B^
	Pulp	42.1 ± 0.6 ^C^	23.0 ± 1.2 ^D^	1.16 ± 0.09 ^C^
	Seed	7.5 ± 1.4 ^A^	3.5 ± 0.8 ^A^	0.26 ± 0.01 ^A^
*Berberis mikuna*	Peel	51.6 ± 0.1 ^D^	7.4 ± 0.3 ^B^	1.04 ± 0.09 ^B,C^
	Pulp	42.3 ± 0.8 ^C^	33.6 ± 1.0 ^E^	1.01 ± 0.04 ^B,C^
	Seed	2.1 ± 0.8 ^A^	6.5 ± 0.2 ^B^	0.37 ± 0.14 ^A^
Positive controls (µg/mL)				
Acarbose		25.0 ± 1.0	1.2 ± 0.1	-
Orlistat		-	-	0.08 ± 0.01

Values are expressed as means ± standard deviation from triplicate measurements. Different letters within the same column indicate statistically significant differences in the effect of polyphenols on enzyme activity across all samples, according to Tukey’s multiple comparison tests (*p* < 0.05). IC_50_: concentration of polyphenolic extract necessary to inhibit 50% of enzyme activity.

**Table 7:** Mutagenicity assays of seed, pulp, and peel powder extracts of *B. burruyacuensis* and *B. mikuna*.

Sample	Powder Extract	Treatment (μg GAE/Plate)	MR TA98	MR TA100
*Berberis burrayucuensis*	Peel	500	1.0	0.8
		250	0.9	0.6
		125	0.7	0.7
	Pulp	500	0.8	0.8
		250	0.8	0.7
		125	0.6	0.8
	Seed	500	1.0	0.6
		250	0.9	0.6
		125	0.7	0.6
*Berberis* *Mikuna*	Peel	500	0.8	0.6
		250	1.2	0.6
		125	0.9	0.7
	Pulp	500	0.8	0.8
		250	0.8	0.8
		125	0.7	0.8
	Seed	500	0.6	0.9
		250	0.9	0.7
		125	0.9	0.8
Positive control			40.37	4.64

MR: mutagenicity relation. Positive control: 4-nitro-phenylenediamine.

## Data Availability

Data is contained within the article.
